# “Bright’s Disease of the Kidneys,” Essentially and Primarily a Blood Disease

**Published:** 1850-03

**Authors:** 


"*Bright's Disease of the Kidneys,*" *Essentially and Primarily a Blood Disease.* By Prof. Walshe, University College, London.

Prof. Walshe observes that the natural history of "Bright's Disease," affords a tolerably sure evidence that it is not primarily a local affection, but originates in the blood. The following are some of the principal facts which support such an opinion:

1. There is no positive and constant naked-eye peculiarity, in the anatomical conditions of the kidney, in all cases where there had been discharge of albuminous urine, and cellular dropsy during life. So true is this, that the most practised observers would hesitate, (and have actually hesitated), from the inspection of *certain* kidneys, to say whether the case had, during life, been attended with the symptoms of simple chronic nephritis, or of "Bright's disease." Of course, this difficulty of distinction is not common, but it exists with quite sufficient frequency to justify the general proposition, as just stated.

2. Neither is there any microscopic character, always present, positively and unfailingly distinctive between the kidneys of persons who have suffered from simple chronic nephritis, or from "Bright's disease." We have frequently, in this hospital, seen clogging of the tubules with simple exudation, (in the stage of "induration matter,") in victims of the latter disease; whilst in others, whose urine had been non-albuminous and alkaline, and in whom no secondary diseases existed, (simple chronic nephritis), fat existed, in some abundance, along with the same "induration matter."

3. There is no positive and direct relationship in cases of "Bright's disease," between the amount of albuminous impregnation of the urine, and of renal disorganization.

4. There is no positive and direct relationship between the number and severity of the "secondary diseases," and of renal disorganization.

5. The urine may, in "Bright's disease," be occasionally copiously albuminous, without co-existing dropsy; or the patient may be dropsical, while his urine is free from albumen.

6. Albuminous impregnation of the urine is caused by other forms of renal disease proper, besides those met with in "Bright's disease," and also by certain morbid states of the blood.

7. Albumen may totally disappear from the urine, *pro tempore*, in the most serious cases of "Bright's disease," yet it cannot be supposed that the physical conditions of the kidney, favoring filtration of serum through the vessels, have suddenly and completely changed.

8. The reason why there is greater clogging of the tubules (general, or limited and "granular,") in the advanced stages of "Bright's disease," (with proto-plastic, or fatty matter, or both), is, that longer time has been allowed for the morbid blood-elements to filtrate through the walls of the vessels. These elements, in part stagnating within the tubules, in part exuded amid the intertubular tissue, accumulate in the renal texture, instead of all passing away with the urine. These elements, deposited granularly, and by uniform infiltration, are the *effects* of the disease, not its *cause*. But as the alterations of the blood are not equable and uniform in their advancement, there is no direct ratio between the amount of clogging of the tubules, &c., and the severity of the disease, considered in the sum of its general manifestations. So true is this, that (as is clinically well known) the very highest amount of "secondary affections" may exist at the outset (anatomically considered) of the disease of the kidney.

9. From the very outset of "Bright's disease," the existence of blood-changes is certain; they are more marked in degree, and earlier in devel. opement, (as appreciable conditions) than in any recognized chronic blood disease.

10. "Bright's disease" has several of the characters of a primary chronic blood disease. Thus, there is no direct ratio between the anatomical changes and the mass of morbid condition constituting the disease; "Bright's disease" tends to the production of various secondary and dependent affections; and the character of these secondary affections partakes of that of the primary (that is, primary in the train of organic changes of the solids) renal changes; they, too, are marked by transudations of serum, and deposition of proto-plastic matter and fat.

11. It may be said, that as urea has been found in the blood at the commencement of acute attacks of "Bright's disease," there is evidence of organic disease of the kidney *ab initio*. But no; functional inactivity is enough to ensure accumulation of urea in the blood; in cholera, there is no textural alteration of the real substance. The true explanation seems to be, that the state of the blood prevents the kidney from acting properly on the elements it is accustomed to excrete, not that its own functional aptitude is at the outset seriously impaired, in other words it is probable that at the commencement the renal cells are still quite able to separate urea, if healthily constituted blood were offered to them by the vessels. This view is, doubtless, hypothetical; but it harmonizes with the well-known and striking fact, that urea may, and does, not uncommonly, disappear from the blood when the anatomical changes in the kidney are carried to extremes, and the case (though a temporary improvement has occurred) is fast tending to a fatal issue. According to the doctrine which regards the accumulation of urea in the blood as the consequence solely of the changes in the kidney, how is this to be explained? According to this doctrine, the effect of a certain cause is decreased as the intensity of that cause is increased.

12. With the progress of deposition within the kidney, the excretory functions of the organ, become, partly mechanically, partly dynamically, interfered with, and hence the blood becomes *secondarily* diseased also.

13. It may be objected that if "Bright's disease" be not essentially a renal, but a blood-disease, *ab initio*, it does not seem clear why the kidney should be the organ on which textural changes are earliest and constantly inflicted. The objection is valueless, until it be explained why tubercle selects the bronchial glands at one period of life for its main nidus, the lungs at another; why cancer chiefly infests the mamma and uterus, &c. *As soon* as the predilections of blood-diseases for certain organs (or the attracting quality of certain organs for special diseased circulating elements) are explained in the instances *inter alia* of tubercle and cancer, the attraction of the morbid material, present in the blood in "Bright's disease," for the kidney will probably be found simple enough. Meanwhile, I would observe, that a fact, which may not be without its application in this matter, was ascertained some years ago by Simon, from the comparative analysis of the blood of the aorta, and of the renal veins in a horse. The quantity of albumen in the former to that in the latter, was found to be as 425 : 446. This natural tendency to excess of albumen in the blood of the renal veins may, perhaps, be in some way connected with its tendency to escape by the channel of the kidney in "Bright's disease." The urine (according to some chemists) naturally contains albumen, but in a proportion so minute that it is undiscoverable by ordinary tests.

From these facts and arguments it appears to me that the following conclusions may fairly be drawn:

"That 'Bright's disease' is a blood-disease, *ab initio*, essentially tending to chronicity, though (like cancer and phthisis) sometimes running an acute course. That the existence of that *ab initio* change follows from: 1. Direct analysis. 2. The general accordance of the pathology of the disease with that of chronic blood-diseases. 3. The inadequacy of the local renal states to account for the local renal phenomena, still less for the general phenomena. 4. The total want of harmony between the renal changes and the general implication of the system, and between the renal changes and the local functional disorders. 5. The facility with which that want of harmony is explicable on the hypothesis of blood-disease. That the precise nature of the primary blood-change is unknown: at present the main peculiarities ascertained (excess of serum of low specific gravity, excess of fat, excess of urea, and deficiency of albumen) are, in all probability, secondary. That the renal textural changes are not the cause of the sum of morbid states, included under the name of "Bright's disease;" and that they do not even furnish a *measure of the intensity* of that disease. That in reality the renal textural changes form at the most the *anatomical character* of the disease, (and their claim in this respect even is not yet fully mace out under all circumstances, *vide* propositions 1 and 2), just as tuberculous deposit in the lung constitutes the anatomical character of phthisis. That the primary blood-changes are the result, probably, of error in the primary and secondary digestion processes. The toxic condition of the blood, whatever it is, is not derived from influences acting from without, but generated within the frame through imperfect organic actions.

That, as in all diseases of this class, (rheumatism, gout, tubercle, cancer, &c.) there is a mysterious constitutional *predisposition* to those imperfect organic actions; without this, all the exciting and immediate alleged causes of 'Bright's disease' fail in the power to evolve it."—*Lancet, July* 14, 1849,

